# Fixed Intelligence Mindset, Self-Esteem, and Failure-Related Negative Emotions: A Cross-Cultural Mediation Model

**DOI:** 10.3389/fpsyg.2022.852638

**Published:** 2022-05-20

**Authors:** Éva Gál, István Tóth-Király, Gábor Orosz

**Affiliations:** ^1^Evidence-Based Assessment and Psychological Interventions Doctoral School, Babeş-Bolyai University, Cluj-Napoca, Romania; ^2^Substantive-Methodological Synergy Research Laboratory, Department of Psychology, Concordia University, Montreal, QC, Canada; ^3^Unité de Recherche Pluridisciplinaire Sport Santé Société, Laboratoire SHERPAS, Université d’Artois, Arras, France

**Keywords:** emotions, failure, fixed intelligence mindset, self-esteem, cross-cultural

## Abstract

A growing body of literature supports that fixed intelligence mindset promotes the emergence of maladaptive emotional reactions, especially when self-threat is imminent. Previous studies have confirmed that in adverse academic situations, students endorsing fixed intelligence mindset experience higher levels of negative emotions, although little is known about the mechanisms through which fixed intelligence mindset exerts its influence. Thus, the present study (*N*_*total*_ = 398) proposed to investigate self-esteem as a mediator of this relationship in two different cultural contexts, in Hungary and the United States. Structural equation modeling revealed that self-esteem fully mediated the relationship between fixed intelligence mindset and negative emotions. Furthermore, results of the invariance testing conferred preliminary evidence for the cross-cultural validity of the mediation model. These findings suggest that, students adhering to fixed intelligence beliefs tend to experience greater self-esteem loss when experiencing academic failure, which leads to higher levels of negative emotions.

## Introduction

A growing number of studies indicate that intelligence mindset (i.e., the way intelligence is conceptualized, malleable vs. unchangeable) exert a considerable impact students’ emotion (Robins and Pals, 2002; Schleider et al., 2015). However, our understanding of the nature of this relationship is scant. Studies show that, beside experiencing overwhelming negative emotions in situations when meeting goals is unattainable (Robins and Pals, 2002), individuals with fixed intelligence mindset (i.e., those believing that intelligence cannot be improved) also tend to report decreases in their self-esteem (e.g., “*Basically I think my GPA sucks, ergo I suck*.”; Robins and Pals, 2002, p. 313; Niiya et al., 2004). Given that drops in state self-esteem is proved to be a precursor of negative affect (Kernis et al., 1989; Crocker and Park, 2004), the present study aimed to explore state self-esteem as a mediator of the relationship between fixed intelligence mindset and negative emotions associated with academic failure.

### Fixed Mindset and Negative Emotional Reactions

Previous studies have demonstrated a consistent link between students’ intelligence mindset and affective states; fixed intelligence mindset being positively associated with negative emotions in cross-sectional (Chan, 2012; King et al., 2012; Wasylkiw et al., 2020) and longitudinal (Da Fonseca et al., 2008; King, 2016) and daily diary studies as well (Baer et al., 2005). Mindset exerts its greatest impact in situations where ego-threat is salient (Blackwell et al., 2007), for example during difficulties, failures and challenges. And studies have documented the adverse affective reactions of individuals with fixed intelligence mindset when encountering failures (Smiley et al., 2016; Tuckwiller and Dardick, 2018). Moreover, a daily diary study found that students holding fixed intelligence mindset perceived academic stressors as threats and experienced a more prolonged stress responses (Lee et al., 2019). Furthermore, teaching students the concept of growth mindset led to an increase in the enjoyment of learning and related activities (Aronson et al., 2002). The association between intelligence mindset and students’ affective states is further substantiated by meta-analyses indicating that fixed intelligence mindset is linked to negative affective states (Gál and Szamosközi, 2016) and poorer mental health outcomes, such as maladaptive perfectionism, symptoms of anxiety and depression (Burnette et al., 2013; Schleider et al., 2015).

### One Potential Mediator Between Fixed Mindset and Affective States: Self-Esteem

Although the association between fixed intelligence mindset and affective states seems meaningful, little is known about the nature of this relationship. Does fixed intelligence mindset influence students’ emotions directly or is its effect mediated by other factors? Burnette et al. (2013) suggested that the relationship between intelligence mindset and behavioral outcomes is complex, where mediator variables might also come into play, so we might assume that mediator variables might be present in the case of affective outcomes as well. Considering that individuals holding fixed intelligence mindset interpret failure and difficulty situations in a manner where failures are overgeneralized to one’s whole identity (e.g., *“I failed because I am incompetent, I am no good for anything.”*; Robins and Pals, 2002) self-esteem could be a potential mediating mechanism between these two constructs. Self-esteem was consistently associated with diverse mental health-related outcomes (Beck et al., 2001; Baumeister et al., 2003; Orth et al., 2012; Bum and Jeon, 2016) and it was frequently found to mediate the impact of different variables on affective states (Shi et al., 2015; Kapikiran and Acun-Kapikiran, 2016; Li et al., 2018). Moreover, numerous studies have found that individuals endorsing fixed intelligence mindset generally report lower levels of self-esteem [Robins and Pals, 2002; Lee et al., 2017; Wasylkiw et al., 2020; for a meta-analysis see Conigrave et al. (2019)].

Since fixed intelligence mindset lies on the assumption that one was born with a fixed amount of intelligence that cannot be improved (Hong et al., 1999) failure becomes not only an indicator of their actual accomplishment (e.g., “I failed this test.”) but their inadequacy as well (e.g., “I am stupid.”; Dweck, 2008). In consequence, individuals with fixed intelligence mindset tend to be preoccupied with demonstrating their abilities (Robins and Pals, 2002), and their self-esteem becomes contingent on external validation (Molden and Dweck, 2006). Research has demonstrated that failure in a domain of contingency leads to drops in state self-esteem, which in turn leads to negative emotional consequences (Crocker and Wolfe, 2001). Correspondingly, several studies have documented that there are fluctuations in fixed mindset individuals’ self-esteem in response to failures. For instance, after an intellectual failure, students with fixed intelligence mindset questioned their global self-worth (Zhao and Dweck, 1997), started to doubt their abilities (Licht and Dweck, 1984), and reported significant decreases in their self-esteem (Niiya et al., 2004). Moreover, a daily diary study has also demonstrated that the impact of daily academic difficulties on self-esteem was moderated by fixed intelligence mindset; those with higher levels of fixed intelligence mindset reported greater drops in their self-esteem when encountering difficulties, suggesting that their self-esteem was more responsive to difficulty experiences. In addition, although mindset was unrelated to students’ daily emotions, they showed a strong association with daily self-esteem, which might suggest that self-esteem is a more proximal determinant of daily emotions than mindset (Gál et al., 2020).

Similarly, previous studies have documented that self-esteem influences individuals’ emotional reactions to failures and negative events (Kernis et al., 1989; Campbell et al., 1991), and that fluctuations in state self-esteem exert a considerable influence on emotions; boosts in self-esteem leading to increases in positive emotions, while drops to increases in negative ones (Crocker and Wolfe, 2001; Crocker and Park, 2004). Given that the self-esteem of individuals with fixed intelligence mindset is highly contingent on external validation and that achievement situations are interpreted in the light of their repercussion to the self and its worth (Dweck, 1999), we might presume that experiencing failures (i.e., failing to validate one’s self-worth) might lead to decreases in state self-esteem, which in turn give rise to negative affective responses. Thus, we hypothesized that in failure situations the impact of fixed intelligence mindset on negative emotions is mediated by their state self-esteem.

### Putting the Mediating Role of Self-Esteem Into Cultural Context

Cross-cultural studies have demonstrated that both the level of self-esteem (Heine et al., 1999) and its impact on mental health varies across cultures, and it is more influential in individualistic cultures (Diener and Diener, 1995). Since individualistic cultures view the self as autonomous and separate from others and their members are encouraged to demonstrate their uniqueness through self-enhancement strategies, self-esteem and positive self-regard become more central. In individualistic cultures there is a huge emphasis on internal abilities and on realizing one’s goals, while in more collectivistic cultures the self is viewed as connected to others and the maintenance of interpersonal relationships is more accentuated which often manifests in self-effacement (Markus and Kitayama, 1991; Tsai et al., 2001). Thus, regarding our proposed mediational model it is possible that the mediating role of self-esteem is not equivalent across cultures.

Regarding intelligence mindset, Dweck (1999) posits that the more individualistic the culture is, the more probable that individuals will cultivate fixed intelligence mindset. These assumptions can be supported by research results demonstrating that in collectivistic cultures there is a focus on the process, and effort is highly valued; features which are consistent with a malleable view of intelligence. Moreover, previous research indicated that there are intercultural differences in effort beliefs (Li, 2012), the value of hard work (Sebestyén et al., 2017), or achievement goals (Stephens et al., 2010), constructs that are closely linked to mindset. Some authors also suggest that the impact of intelligence mindset on achievement might not be universal across cultures (Clegg et al., 2017). Given these results we might expect cross-cultural variation in the level of fixed intelligence mindset. Moreover, since in individualistic cultures demonstrating one’s uniqueness and maintaining a positive self-regard is fundamental, which is also a major preoccupation of individuals with fixed intelligence mindset (Robins and Pals, 2002) it is possible that the effect of fixed intelligence mindset on self-esteem might also vary across different cultural contexts.

However, as far as we are aware, no cross-cultural comparison was conducted to test whether the associations between fixed mindset, self-esteem, and negative emotions are comparable across cultures. Thus, the present study aimed to investigate the comparability of the proposed mediational model in two different cultural contexts (i.e., Hungary and the United States). While the United States has one of the most individualistic cultures, Hungary has a dual character where, although individualism is more dominant, Western and Eastern features are equally present (Holicza, 2016). Given these cultural differences among Hungary and the US and the culture-specific role of self-esteem and possibly of intelligence mindset, it is worthwhile to explore possible cross-cultural similarities or differences.

### The Present Study

The present study proposed to investigate self-esteem as a potential mediator between fixed intelligence mindset and negative emotions. Furthermore, it also aimed to test the validity of the proposed mediational model across cultures. It was hypothesized that fixed intelligence mindset would be positively associated with failure-related negative emotions and negatively with failure related self-esteem, and that self-esteem would mediate the impact of mindset on negative emotions. The analysis regarding the intercultural invariance of the mediational model was conducted in an exploratory manner, with no specific hypothesis formulated.

## Methods

### Participants

The present study was based on two college student samples from two different countries. Sample 1 consisted of 194 Hungarian college students (126 women) aged between 18 and 30 years (M_*Sample* 1_ = 22.50, SD_*Sample* 1_ = 2.94). The Hungarian sample mainly included undergraduate (*N* = 161) students with diverse majors; the most represented being physical education and coaching (22%), psychology (14%), computer (10%) and educational sciences (6%), linguistics (5%) and health sciences (5%). Sample 2 consisted of 204 US college students (146 women), aged between 18 and 30 years (M_*Sample* 2_ = 21.57, SD_*Sample* 2_ = 2.28). The US sample included only undergraduate students with diverse majors like psychology (11%), biology (10%), international affairs (7%), anthropology (6%), linguistics (6%), computer sciences (5%), and economics (5%).

### Measures

#### Intelligence Mindset

Participants’ intelligence mindset was assessed by the fixed mindset subscale of the Implicit Theories of Intelligence Scale (Dweck et al., 1995; Orosz et al., 2017). The subscale consists of four items capturing the belief in the unchangeability of one’s intelligence (e.g., “*I do not think I personally can do much to increase my intelligence*.”). Participants had to express their agreement on a 6-point scale (1 = completely disagree; 6 = completely agree), higher scores indicating stronger fixed intelligence mindset. The scale showed good internal consistency (αUS = 0.91; αHU = 0.90).

#### Failure-Related Self-Esteem

Self-esteem was measured using the Rosenberg Self-Esteem Scale (Rosenberg, 1965; Sallay et al., 2014). Participants were asked to recall their latest academic setback and indicated on a 5-point scale (1 = completely disagree; 5 = completely agree) the extent to which they would have agreed with each statement in that situation (e.g., *“I think I am able to do things as well as most other people.”*). Negatively worded items were reverse coded, higher scores reflecting higher self-esteem. The scale showed good internal consistency (αUS = 0.89; αHU = 0.87).

#### Failure-Related Negative Emotions

Negative emotions were assessed using Pekrun et al. (2011) list of negative academic emotions, which typically arise during learning and related activities. Based on their latest academic setback, participants indicated on a 5-point scale (1 = very slightly or not at all; 5 = extremely) the extent to which they have experienced specific negative emotions (i.e., anxiety, anger, shame, disappointment, hopelessness) during this event. The internal consistency of the scale was adequate (αUS = 0.83; αHU = 0.84).

### Procedure

Participants were recruited through advertisements in online groups frequented by the students of one of the major universities in Hungary and Georgia, United States. The study was conducted in accordance with the Declaration of Helsinki and was approved by the ethical board of the first author’s university; participants’ informed consent was also obtained. Participation was completely voluntary and anonymous and it consisted of completing a set of online questionnaires. After completing the demographic questions and the intelligence mindset scale, participants were instructed to recall as vividly as they could the last time, they have experienced academic failure. Subsequently, based on these recalled experiences, they completed the self-esteem and affective measures.

### Statistical Analysis

Statistical analyses were conducted using Mplus 8 (Muthén and Muthén, 2017); the robust maximum-likelihood (MLR) estimator was used which provides fit statistics and standard errors that are robust to the non-normality of the data. Preliminary measurement models were estimated to verify the psychometric properties of the scales using a confirmatory factor analytic (CFA) model with fully latent variables, which provides a way to explicitly take measurement errors into account (Finkel, 1995), thus leading to more accurate parameter estimates. *A priori* correlated uniquenesses were included between a subset of items belonging to the self-esteem factor to account for their negative-wording effect (Marsh et al., 2010, see also in Supplementary Material).

Tests of measurement invariance using a structural equation modeling framework with multi-group CFA, were conducted with the gradual addition of equality constraints on various parameters (Milsap, 2011): configural invariance (same factor structure), weak invariance (equal factor loadings), strong invariance (equal intercepts), strict invariance (equal uniquenesses), as well as the invariance of correlated uniquenesses, the latent variance-covariance matrix, and the latent means (for more information see Supplementary Material).

The following goodness-of-fit indices were used to evaluate the adequacy of the models (Hu and Bentler, 1999; Marsh et al., 2005): comparative fit index (CFI; ≥0.95 for excellent, ≥0.90 for adequate), Tucker- Lewis index (TLI; ≥0.95 for excellent, ≥0.90 for adequate), root-mean-square-error of approximation (RMSEA; ≤0.06 for excellent, ≤0.08 for adequate) with its 90% confidence interval. For purposes of model comparisons, relative changes (Δ) in the fit indices were compared with a change of at least 0.010 for CFI and TLI and a change of at least 0.015 for the RMSEA were taken to suggest meaningful differences (Cheung and Rensvold, 2002; Chen, 2007).

The most invariant measurement model was used to test the proposed predictive model in three steps. First, a partial mediation model was estimated in which fixed mindset predicted self-esteem and emotion while self-esteem also predicted negative emotions. In the second step, the direct path between fixed mindset and negative emotions was removed. In the third step, the invariance of the predictive paths was tested by constraining the regressive paths to equality across the two samples (Tóth-Király et al., 2020). In the final predictive model, in order to assess the mediation hypothesis, 95% bias-corrected bootstrapped confidence intervals were also computed in Mplus with the maximum likelihood estimator as bootstrapping is not available with MLR (Schellenberg et al., 2016; Tóth−Király et al., 2019). Based on Preacher and Hayes (2008), 5,000 bootstrap replication samples were requested.

## Results

Model fit information for the measurement models is reported in Table 1, and it shows that all models achieved an adequate level of fit. Tests of measurement invariance provided support for the configural (i.e., the investigated constructs are represented in the same way in United States and Hungary) and weak invariance (i.e., items contribute similarly to the latent factors in both samples) of these preliminary measurement models. However, strong invariance (i.e., items loading on latent factors have similar means across groups) was not achieved (ΔCFI = −0.022, ΔTLI = −0.019, ΔRMSEA = +0.007), thus, we tested a partial strong invariant model in which two self-esteem intercepts were freed up. This partial strong model demonstrated adequate model fit changes (ΔCFI and ΔTLI ≤ 0.010; ΔRMSEA ≤ 0.015). Subsequent tests supported the complete invariance of this measurement model up to the level of latent mean invariance which was retained for interpretation and further analyses. The complete invariance of the model indicates that the correlations between the investigated constructs and the group-means of the latent variables are equal in the United States and Hungarian samples.

**TABLE 1:** Invariance testing of the measurement and structural models across countries.

Model	χ^2^ (df)	CFI	TLI	RMSEA	90% CI	Δχ^2^ (df)	Δ CFI	Δ TLI	Δ RMSEA
**Measurement models**									
US sample	244.155* (129)	0.928	0.915	0.066	0.053, 0.079	—	−	−	−
Hungarian sample	217.321* (129)	0.937	0.925	0.059	0.045, 0.073	—	−	−	−
**Measurement invariance**									
Configural	460.823* (258)	0.932	0.920	0.063	0.053, 0.072	—	−	−	−
Weak	477.053* (273)	0.932	0.924	0.061	0.052, 0.070	15.422 (15)	0.000	+ 0.004	–0.002
Strong	554.171* (288)	0.911	0.905	0.068	0.060, 0.077	79.408* (15)	–0.022	–0.019	+ 0.007
Partial strong	517.391* (286)	0.923	0.917	0.064	0.055, 0.072	41.053* (13)	–0.009	–0.007	+ 0.003
Strict	541.974* (304)	0.920	0.920	0.063	0.054, 0.071	27.212 (18)	–0.003	+ 0.003	–0.001
Correlated uniquenesses	543.268* (307)	0.921	0.921	0.062	0.054, 0.071	2.070 (3)	+ 0.001	+ 0.001	–0.001
Latent variance-covariance matrix	555.135* (313)	0.919	0.921	0.062	0.054, 0.071	11.866 (6)	–0.002	0.000	0.000
Latent means	560.947* (316)	0.918	0.921	0.062	0.054, 0.071	5.867 (3)	–0.001	0.000	0.000
**Predictive model**									
Partial mediation free relations	559.279* (313)	0.918	0.920	0.063	0.054, 0.071	-	−	−	−
Full mediation free relations	559.143* (315)	0.918	0.921	0.062	0.054, 0.071	0.083 (2)	0.000	0.000	0.000
Full mediation equilibrium	560.883* (317)	0.918	0.921	0.062	0.054, 0.071	1.622 (2)	0.000	0.000	0.000

*p < 0.05; χ^2^, robust chi-square test of exact fit; df, degrees of freedom; CFI, comparative fit index; TLI, Tucker–Lewis index; RMSEA, root mean square error of approximation; 90% CI, 90% confidence interval of the RMSEA; Δχ^2^, robust (Satorra–Bentler) chi-square difference test (calculated from loglikelihood for greater precision); Δ, change in fit information relative to the previous model.

In general, our results revealed well-defined and reliable factors for fixed mindset (λ = 0.779 to 0.902), self-esteem (λ = −0.568 to 0.783), and negative emotions (λ = 0.641 to 0.795). Latent correlations reflected our *a priori* expectations: self-esteem negatively correlated with fixed mindset (*r* = −0.331, *SE* = 0.058, *p* < 0.001) and negative emotions (*r* = −0.725, *SE* = 0.043, *p* < 0.001), while fixed mindset correlated positively with negative emotions (*r* = 0.229, *SE* = 0.059, *p* < 0.001).

**FIGURE 1:**
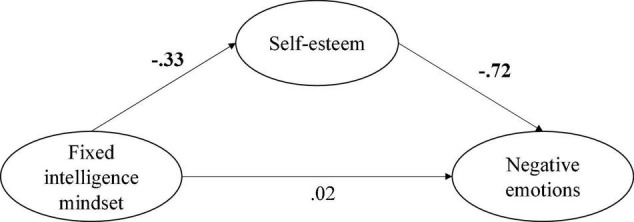
Mediational model. All variables presented in ellipses are latent variables. For the sake of simplicity measured variables are not depicted in this figure. One-headed arrows represent standardized regression weights. Significant regression weights are in bold.

The model fit results from the predictive models are reported in the bottom section of Table 1, and show that the partial and full mediation models have virtually identical fit indices. Coupled with the fact that fixed mindset did not statistically significantly predict negative emotions, we decided to retain the full mediation model. Adding equality constraints to the paths of this predictive model resulted in negligible differences in model fit, suggesting that these predictive paths can be considered equal in the two samples. The examination of the parameter estimates from this model (Figure 1) showed that fixed mindset negatively predicted self-esteem (β = −0.330, *SE* = 0.057, *p* < 0.001), while self-esteem also negatively predicted negative emotions (β = −0.725, *SE* = 0.044, *p* < 0.001). Mediation analyses revealed that the indirect relation between fixed mindset and negative emotions was statistically significant (indirect β = 0.239, CI = 0.157 to 0.326, *p* < 0.001), indicating that self-esteem fully mediated mindsets’ impact on negative emotions. Finally, the proportion of explained variance was 10.9% for self-esteem and 52.5% for negative emotions.

## Discussion

The results of the present study supported that there is a link between fixed intelligence mindset and failure-related negative emotions. However, we have also found that fixed intelligence mindset is related to negative emotions only indirectly as failure-related self-esteem fully mediated mindset’s effect. This suggests that the belief in the unchangeable nature of intelligence primarily affects one’s self-esteem (e.g., “I was born with a fixed amount of intelligence, so failing means that I do not have enough intellectual capacities to succeed so I am incompetent/less adequate.”), which in turn would lead to more negative affective outcomes (e.g., shame, hopelessness, disappointment). This finding is not surprising as Dweck (2008) theory posited that individuals adhering to fixed intelligence mindset tend to attribute achievement to internal factors (i.e., unchangeable innate abilities) and interpret achievement situations in terms of how these situations reflect on themselves and their intelligence. Consequently, their self-esteem becomes more contingent on external validation, and proving one’s intelligence turns into a central preoccupation. Moreover, failures or setbacks represent a threat to one’s self-worth as they might reveal one’s inadequacies or shortcomings. Given this meaning system where performance has direct implications to one’s self and its worth, it is understandable why students endorsing fixed intelligence beliefs reported lower self-esteem related to a recalled failure experience. Similarly, Crocker and Park (2004) have also suggested that failures in domains on which self-esteem is contingent lead to drops in self-esteem and increases in negative emotions. Moreover, studies indicated that following self-esteem loss, feelings of anger and shame are also common reactions (Tangney, 1999). Fluctuations in self-esteem were also linked to fluctuations in optimism, anxiety, and perceived control over events (Gable and Nezlek, 1998).

Although studies on intelligence mindset and its association with affective states have been conducted in different cultures, to the best of our knowledge, no cross-cultural investigation has been carried out to test whether these associations and effects are similar in different cultures. Results of the invariance testing suggested that the investigated constructs have the same structure and meaning in different groups, so between-group comparisons are feasible. Moreover, results also indicated that there were no differences in the relationship between the study variables, in the strengths of these relationships, or in the group means across United States and Hungarian samples. Furthermore, the examined mediational model proved to be consistent across different cultural settings. Although cross-cultural research generally agrees that self-esteem might not equally determine positive and negative outcomes in all cultures (Farruggia et al., 2004); feeling good about oneself being more critical in western individualistic cultures (e.g., United States); the present study revealed that self-esteem exerts comparable effects on students’ negative emotions across the United States and Hungarian samples. The comparable role of self-esteem in determining students’ failure-related emotions could be explained by the fact that, although Hungary, compared to the United States, has a less individualistic culture, it combines Western and Eastern features alike (Falkné Banó, 2014). Thus, it is possible that in this context, achievement not only represents a way to demonstrate one’s uniqueness, but also a way to meet social norms. Thus, feeling good or bad about oneself after experiencing failure, although for different reasons (i.e., demonstrating uniqueness and individual ability vs. meeting familial or social standards), equally determines individuals’ emotional reactions.

Previous studies have documented that failures and difficulties are emotionally taxing experiences for individuals holding fixed intelligence mindset, as they tend to exhibit helpless reactions (Dweck and Yeager, 2019). If future studies would experimentally reinforce our results, that would point out two possibilities for intervention. First of all, intelligence mindsets can be changed through carefully designed interventions (Yeager et al., 2019). Providing the lens of growth mindset allows students to view academic difficulties and failures as less threatening situations to their self-worth. As the result of their changed mindset beliefs, their self-esteem might become less responsive to academic adversities, which in turn would promote the emergence of more adaptive emotional experiences. If students recognize that through learning and practice their abilities are able to develop, failures and difficulties would be less decisive experiences. Acknowledging the role of effort in determining performance might help students avoid interpreting performance as the direct reflection of one’s innate intellectual ability. Thus, failure’s implications in self-esteem would also lessen since it would not pass judgment on one’s unchangeable traits. Not needing to validate one’s intelligence through performance might help students to approach difficulties and failures more adaptively and to focus more on the process of learning rather than on the repercussions of performance on their self-worth.

Secondly, self-esteem is one of the strongest predictors of emotional problems (Leary et al., 1995) and given the role of self-esteem in mediating fixed mindset’s effect on negative emotions, intervening at the level of self-esteem by employing mindset intervention and cognitive behavioral therapy (CBT) techniques could not only ameliorate fixed mindset’s impact on failure-related emotions, but it could also benefit students’ mental health. Fixed mindset “transforms failures from an action (e.g., “I failed.”) to an identity” (e.g., “I am a failure.”; Dweck, 2008, p. 33), which according to CBT involves cognitive distortions like labeling or overgeneralization. So, teaching students to evaluate the evidence-base and logical correctness of their thoughts and to formulate alternative rational ones, might improve the way students cope with failures. Moreover, according to Ellis et al. (2010), self-esteem could be conceptualized as a global evaluation of one’s self, which given the complexity and ever changeability of the self, is an unscientific overgeneralization. Thus, instead of pursuing high self-esteem, adopting unconditional self-acceptance, would be more constructive, since in this case the evaluation of the self is not contingent on external factors. So, we might speculate that teaching students to refrain from making global self-evaluations, to separate the evaluation of their performance from the evaluation of their self might make students’ self-esteem to be more resilient in the face of academic adversities. Moreover, unconditional self-acceptance was associated with higher achievement (Balkis et al., 2013) and positive mental health outcomes (Chamberlain and Haaga, 2001).

### Limitations

Although the present study offers new and meaningful insights regarding the relationship between intelligence mindset, self-esteem, and affective states, they are rather preliminary in nature, due to the various limitations of the present study. First of all, the size of the two samples was relatively small, and they mainly consisted of female college students, thus limiting the generalizability of the results. Moreover, results are based on self-reported and cross-sectional data, which are susceptible to social desirability bias and does not allow the investigation of causality. The affective and self-esteem measures were completed based on recollections of past failures; thus, it is unknown whether our measurements properly reflect emotions emerging during real-life failure experiences. Future studies should investigate this mediational model in experimental settings as well. Furthermore, in the present study, academically contingent self-worth was not assessed, it might thus be possible that the strength between fixed intelligence mindset and failure-related self-esteem might differ according to how heavily one’s self-worth is staked on academic performance.

## Data Availability Statement

The raw data supporting the conclusions of this article will be made available by the authors, without undue reservation.

## Ethics Statement

The studies involving human participants were reviewed and approved by Comisia de etica Universitatea Babes-Bolyai. The patients/participants provided their written informed consent to participate in this study.

## Author Contributions

All authors contributed to the study design, literature review, data gathering, manuscript writing, and to the data analyses and interpretation, commented on the draft, contributed to the final version, approved the publication of the manuscript, and agreed to be accountable for all aspects of the work.

## Conflict of Interest

The authors declare that the research was conducted in the absence of any commercial or financial relationships that could be construed as a potential conflict of interest.

## Publisher’s Note

All claims expressed in this article are solely those of the authors and do not necessarily represent those of their affiliated organizations, or those of the publisher, the editors and the reviewers. Any product that may be evaluated in this article, or claim that may be made by its manufacturer, is not guaranteed or endorsed by the publisher.
