# Supplementary material for: Fixed Intelligence Mindset, Self-Esteem, and Failure-Related Negative Emotions: A Cross-Cultural Mediation Model
Source: Front Psychol. 2022 May 20;13:852638. doi: 10.3389/fpsyg.2022.852638 (PMC9165622; doi:10.3389/fpsyg.2022.852638)
Supplement: Supplementary file 1 [file Data_Sheet_1.docx]

**Supplementary Materials**

**1. Including a-priori uniqueness between negative worded items of the Rosenberg Self-Esteem Scale**

Research suggests that positive and negative wording of the items could lead to systematic measurement error (i.e., a systematic variance in the responses induced by the method which was used to measure the variable; method effect; Chen et al., 2007). The method effect might bias analysis and results (i.e., factor structure and scale reliability of the scale; Horan et al., 2003), and it is considered to be larger in the case of negatively worded items (Chen et al., 2007). Due to the method effect, unidimensional constructs might appear multidimensional, positively and negatively worded items loading on different factors (Chen et al., 2007).

The method effect due to wording was extensively explored in the case of the Rosenberg Self-Esteem scale. This scale assesses global self-esteem and it contained 5 positively and 5 negatively worded items. Studies exploring its factor structure evidenced the presence of two factors, one encompassing the negatively worded items and the positively worded ones, and the authors concluded that the presence of two factors is due to different item phrasing (Carmines & Zeller, 1979; Marsh et al., 2010; Quilty et al., 2006). In order to compensate the method effect, one of the alternative measurement models is a model with one-factor and correlated uniqueness for the negatively worded items (Chen et al., 2007). Thus, in order to counter the impact of method effect we included a priori correlated uniqueness for the negatively worded items.

**2. Measurement invariance testing**

Before investigating the associations between the variables of interest (e.g., fixed intelligence mindset, self-esteem, and negative emotions), we verified the equivalence and the comparability of the constructs across the two samples via tests of measurement invariance using a structural equation modeling framework with multi-group confirmatory factor analysis.

Tests of measurement invariance ensure the psychometric equivalence of a construct across groups and that the same constructs have the same structure or meaning in different groups, which is a prerequisite for comparing groups and relationships across constructs in different groups (Millsap, 2011; Putnik & Bornstein, 2016).

Tests of measurement invariance have seven levels and the invariance of each level is tested by imposing equality constraints on different parameters. Configural invariance investigates whether constructs are represented in the same way in different groups and whether they have the same factor structure. Weak invariance explores whether each item contributes to the latent factor in the same manner in each group (i.e., metric invariance; Putnik & Bornstein, 2016). Weak invariance is tested by constraining the factor loadings to be equal across the groups. Strong invariance (i.e., scalar) is tested by constraining the item intercepts to be equal across groups. If scalar invariance is met that implies that the means of the items loading on latent variables are equal across groups and latent means can be compared (Putnik & Bornstein, 2016). Strict invariance (i.e., residual) refers to the fact that the unique variance of the items unrelated to the factor (i.e., item residual) and the error variance (i.e., measurement error) are similar across the two groups (Putnik & Bornstein, 2016), which suggest that items measure the underlying constructs with the same precision which enables comparisons based on manifest scores (Tóth-Király & Neff, 2020). The invariance of the latent variances and covariances explores similarities in correlations among the constructs, while the invariance of the latent means indicates whether between-group differences are present based on group means on the latent factors (Tóth-Király & Neff, 2020).

Although only weak invariance is needed to test associations between latent variables across groups (Tóth-Király et al., 2020, p.2988), pursuing additional tests of invariance has important statistical advantages such as having a more parsimonious model and obtaining more stable and trustworthy estimates.

The equivalence of the relationship between study variables across groups was tested by constraining the paths of the predictive model to be equal across groups.

In order to establish if the different invariance levels were achieved, relative changes (Δ) in the fit indices were compared between two subsequent invariance levels. A change of at least .010 for CFI and TLI and a change of at least .015 for the RMSEA were taken to suggest that by constraining a parameter significant model fit changes have occurred, which indicates that the specific invariance level is not supported (Chen, 2007; Cheung & Rensvold, 2002).

**References for Supplementary Materials (references which are not included in the manuscript)**

Carmines, E. G., & Zeller, R. A. (1979). *Reliability and validity assessment*. Beverly Hills, CA: Sage.

Chen, Y-H., Rendina-Gobioff, G., & Dedrick, R. F. (2007, 14-16 November). Detecting effects of positively and negatively worded items on a self-concept scale for third and sixth grade elementary students [Paper presentation]. Annual Meeting of the Florida Educational Research Association, Tampa, Florida.

Corwyn, R. F. (2000). The factor structure of global self-esteem among adolescents and adults. *Journal of Research in Personality.* 34, 357–379. doi: 10.1006/jrpe.2000.2291

Horan, P. M., DiStefano, C., & Motl, R. W. (2003). Wording effects in self-esteem scales: Methodological artifact or response style?. *Structural Equation Modeling*, *10*(3), 435-455.

Putnick, D. L., & Bornstein, M. H. (2016). Measurement invariance conventions and reporting: The state of the art and future directions for psychological research. *Developmental review*, *41*, 71-90. <https://doi.org/10.1016/j.dr.2016.06.004>

Tóth-Király, I., & Neff, K. D. (2021). Is self-compassion universal? Support for the measurement invariance of the Self-Compassion Scale across populations.  *Assessment*, *28*(1), 169-185. https://doi.org/10.1177%2F1073191120926232

Quilty, L. C., Oakman, J. M., & Risko, E. (2006). Correlates of the Rosenberg Self-Esteem Scale method effects. *Structural Equation Modeling, 13*, 99-117.
